# Implementing food bank and healthcare partnerships: a pilot study of perspectives from charitable food systems in Texas

**DOI:** 10.1186/s12889-021-12031-w

**Published:** 2021-11-06

**Authors:** Natalie S. Poulos, Eileen K. Nehme, Molly M. O’Neil, Dorothy J. Mandell

**Affiliations:** Department of Community Health, University of Texas Health Science Center at Tyler, 11937 US Hwy 271, Tyler, TX United States 75708

**Keywords:** Charitable food systems, Healthcare systems, Health partnerships, Food insecurity, Food bank partnerships

## Abstract

**Background:**

Partnerships between charitable food systems and healthcare systems have been forming across the country to support individuals and families experiencing food insecurity, yet little research has focused on these partnerships, particularly from a food bank perspective. The objective of this exploratory pilot study was to identify implementation challenges and facilitators of charitable food system and healthcare partnerships from the food bank perspective.

**Method:**

Texas food banks with existing food bank/healthcare partnerships were identify through website review and support from Feeding Texas. Interview questions were tailored to each interview, but all focused on identify program components of the food bank/healthcare partnership and implementation barriers/facilitators of the partnership. In total, six interviews were conducted with food bank/healthcare partnership leaders (*n* = 4) and charitable food system experts (*n* = 2) about their experiences of working with food bank/healthcare partnerships. All interviews were completed via Zoom and took between 30 and 60 min to completed. Detailed notes were taking during each interview, and immediately discussed with the complete research time to formulate broad implementation themes.

**Results:**

Interviews suggest unique implementation challenges exist at all levels of food bank/healthcare partnerships including the partnership, program, and system levels. Partnership-level implementation challenges focused on issues of partnership scale and data collection, sharing, and analysis. Program-level implementation challenges focused on food and produce expectations. Structural-level implementation challenges included issues of food safety, subsidized food regulations, and patient privacy. Implementation facilitators included leadership support, mission compatibility/organizational readiness, food insecurity training, and identify of partnership champions.

**Conclusions:**

This study adds to the growing interest in food bank/healthcare partnership as it highlights unique implementation challenges and facilitators for cross-sector partnerships between healthcare systems and community-based charitable food systems. Ultimately, we believe that collaborative discussion among leaders of charitable food systems and healthcare systems is needed to overcome outlined implementation challenges to better facilitate sustainable, equitable implementation of food bank/healthcare partnerships.

## Background

The COVID-19 pandemic has exposed the vulnerability of many Americans to food insecurity, meaning that at times they were unable to acquire adequate, nutritious food for the household [1]. In 2018, approximately 11.1% of U.S. households experienced food insecurity [1], yet a recent analysis of the U.S. Census Household Pulse Survey found that rates of food insecurity have doubled overall and tripled among households since the COVID-19 pandemic began [2]. This is problematic as food insecurity associated with a wide range of negative physical and mental health outcomes throughout the life course [3, 4].

The recognition of food security as a critical social determinant of health has led to resolutions from leading health professional organizations including the American Academy of Pediatrics [5] and the American Academy of Family Physicians to promote screening and interventions that address food insecurity [6]. However, some healthcare providers are uneasy about screening for food insecurity because they feel unprepared to make adequate referrals to support programs [7]. To create a strong referral option for healthcare providers and improve the support available to individuals and families experiencing food insecurities, partnerships between food banks and healthcare providers have been gaining interest across the country. These partnerships are often unique and tailored to both the food bank and healthcare partner. Although partnerships are relatively new, Feeding America, the largest national network of food banks in the United States, has begun advocating for the development of food bank/healthcare partnerships as evidenced by the release of two resources that address the importance and need for increased food insecurity screening among healthcare providers, as well as examples of organizational readiness for healthcare partners interested in partnering with food banks [8, 9]. Feeding America suggests that through screening of food insecurity by the healthcare provider and referral to the charitable food system, individuals and families experiencing food insecurity will be more likely to receive food needed to decrease hunger.

Research on food bank/healthcare partnerships has begun to provide insights into the clinic experience and context of these partnerships. For example, four models of partnership have been identified in the literature including partnerships focused on food insecurity screening within the healthcare setting followed by referrals to local food banks and pantries [10–13], in addition to clinic interventions addressing populations with specific disease profiles such as diabetes [14, 15], the establishment of co-located food pantries within clinical settings [16], and food prescription programs [17]. Yet, little research has considered implementation of food bank/healthcare partnerships from a charitable food system perspective and this perspective is needed to inform effective guidance for both food banks and healthcare systems.

The purpose of this pilot study was to explore the challenges and facilitators to implementing partnerships between food banks and healthcare, specifically from the perspective of food banks. This study uses qualitative interviews with charitable food system representatives to outline implementation challenges and facilitators needed to successfully implement and sustain food bank/healthcare partnerships.

## Methods

Food banks with healthcare partnerships were identified through a review of food bank websites in Texas and documentation on Food Bank/Healthcare Partnerships from Feeding Texas, the state-wide organization that supports collaboration across food banks [18]. Of the 21 food banks in Texas, eight food banks were identified as having an active food bank/healthcare partnership in 2020. One additional food bank suspended their food bank/healthcare partnership in 2019. To identify additional implementation challenges, individuals with extensive experience working alongside food banks were identified through Feeding Texas contacts or known professional contacts. Each charitable food system expert was chosen to represent different perspectives and experiences with charitable food systems including state-wide food bank networks and community nutrition expertise. Potential participants were invited by email to participate in virtual interviews. In total, the eight food banks that were identified as having a food bank/healthcare partnership were asked to participate. Two charitable food system experts were requested to participate to provide perspectives of working within the charitable food system.

Questions for each interview focused on identification of food bank/healthcare partnership programs, implementation challenges and facilitators for these partnerships, and sustainability challenges for food bank/healthcare partnerships. Interviews were completed on Zoom by trained research staff and took between 30 min to an hour to complete. Detailed notes were taken during each interview, and immediately reviewed and discussed by the complete research team. Structured interview questions were not asked of each participant nor was the interview recorded to allow for interviews to be based on participants experience and expertise, while simultaneously protecting participants privacy.

Generation of themes was derived through inductive analysis of interview notes and iterative discussion with the complete research team until consensus was reached. All team members have graduate-level education and experience in qualitative methods and analysis. Individual participants are not attributed to thematic content, as the focus of the interview was on organizational implementation. All interviews were completed in June 2020.

Data pertaining to the food bank/healthcare partnership was the only data collected during interviews. After reviewing the Health and Human Services (HHS) guidelines on human subject research, this study was determined to be nonhuman subjects research according to HHS guidelines [19]; therefore, the Institutional Review Board was not needed.

## Results

Overall, eight food banks and two charitable food system experts were contacted to participate in the interviews. A total of six interviews were completed. Four of the food banks contacted did not respond to the participation request. Each food bank participant directly worked within the food bank/healthcare partnership (*n* = 4). Charitable food system experts included individuals with extensive knowledge of food bank networks (*n* = 1) and management of charitable food organizations (n = 1). See the Table 1 for the program scope and experience of participants.

**Table 1:** Descriptive characteristics of food banks and charitable food system experts interviewed (*n* = 6)

Size	Program Scope/Experience
**Food Bank/Healthcare Partnership Leader**	
Large, urban	Food prescriptions, onsite food pantry, mobile food markets, screening, and referral network (Current)
Small, rural	Screening and referral (Current)
Large, urban	Mobile food/produce delivery, therapeutic food clinics, and electronic referral system (Current)
Large, urban	Onsite food pantry, mobile food delivery, and tailored food boxes (*Discontinued*)
**Charitable Food System Expert**	
State-wide network	Leadership in state-wide organization that supports all food banks in Texas (Current)
Regional, academic expert	Academic expert in community nutrition (Current), Leadership in local meal support program (*Previous*)

Responses were organized into implementation challenges at the partnership level, program level, or structure/system level and implementation facilitators for creating sustainable food bank/healthcare partnerships. Each is discussed in below using context from interviews.

### Hyphenate Partnership-level

Participants made it clear that discussions about expected program scale within a food bank/healthcare partnership should be at the center of partnership planning and negotiation. For example, participants indicated that food banks work the best “at-scale,” meaning they function efficiently when they can deliver large quantities of food to communities. Ultimately, they suggest that larger the program scale the better, when it comes to working most efficiently with food banks. Participants went on to suggest that if a food bank is considering partnering with a clinic that serves a small population, it could be challenging or not seen as a priority for the food bank, as outcomes such as pounds of food delivered are the primary outcomes for a food bank.

Another implementation barrier reported by participants centers around data: data collection, data sharing, and program evaluation. All participants suggested that food banks often do not have the capacity or expertise to collect data or enter large amounts of data, nor are food bank staff commonly experienced or trained in data collection, yet they understand that without data to support the program, there is little chance of the program surviving because of funding pressures to produce measurable outcomes. Problematic data sharing was also a common theme within interviews. Specifically, participants noted that even with data agreements between the food bank and the healthcare providers, healthcare partners often did not collect complete data or did not provide timely data reports back to the food bank as outlined in agreements. All participants also mentioned that some clinics may not want to share or have the capacity to share individual level data because of patient privacy concerns. These difficulties with data sharing are seen as a problem for food banks because it is difficult to show program success to current and future funders with incomplete or missing data. Data discussions during interviews almost always ended with a focus on evaluation. To facilitate effective program evaluation, participants emphasized that food banks should think ahead to what metrics and outcomes are essential to allow for tailored data collection across programs and prevent excessive data collection on measures that are not useful.

### Program-level implementation challenges

When entering a partnership between charitable food partners and healthcare providers, multiple participants emphasized the need of clear communication and discussion about food expectations. For example, food banks operate with significant amounts of donated foods, so certain foods cannot be guaranteed. This variability often creates frustration because clinics can have expectations that are unachievable by food banks. Given the known variability in food, food banks should be clear on what it can deliver early in the formation of the partnership to ensure a positive relationship between food bank and healthcare partner.

Another common implementation challenge discussed among participants was an inability of the food bank to receive a variety of produce, a commonly requested item from healthcare partners. Reliable and varied produce is not something that most food banks are able to provide year-round. For example, within a 30-pound box of produce, clients may only get 2–3 types of produce, as it depends on what is in season and available in bulk. Issues of culturally appropriate foods were also discussed, as not all food banks will have access to bulk produce that is culturally appropriate or familiar/easy to cook with for recipients. To help with food and produce consistency, results suggested food banks should consider working with local farms to produce consistent produce.

### Structural challenges

All participants discussed the structural implementation barrier of food safety requirements. Participants reiterated that few healthcare partners have adequate refrigeration to safely store produce or perishable foods. This lack of safe food storage can often result in a limited variety of foods available within food boxes distributed at clinic sites. Expired food presents another structural challenge for food bank/healthcare partnerships as a significant amount of food available in food banks has been donated. When relying on donated food, as all food banks and pantries do, participants suggest the food bank and healthcare partner need to have clear guidelines around how to handle expired food that enters the charitable food system.

Participants also highlighted that many food bank programs are funded through federal and state programs with that carry government regulations that specify on how funding is used. While participants reiterated that food banks have a strong purchasing power for commodity and government foods, food banks are also required to use that purchased food for specific populations. For example, participants noted that food items purchased through government funded programs commonly required recipients to acknowledge income eligibility requirements, which can make distributing food to children particularly complicated because children are unable to legally complete the income verification forms. One exception to the income verification required discussed among participants was when the food was highly perishable (i.e., produce). These highly perishable foods can be distributed to any clinic visitor, regardless of income, to ensure that the produce was distributed quickly.

Given the nature of any healthcare partnership, issues of patient privacy were discussed by participants as a barrier that must be overcome or addressed before entering a partnership. Participants indicated that food bank/healthcare partnerships would likely involve data sharing of sensitive medica data, and therefore, food bank staff should receive training on patient privacy and the Health Insurance Portability and Accountability Act (HIPAA), something that is currently uncommon in food bank staff trainings. Participants also discussed that healthcare partners need to have a similar understanding of protected patient data. Specifically, results highlighted that some healthcare systems see sharing food insecurity screening results with the food banks as a violation of HIPAA, yet other healthcare partners willingly share this information with the food bank. This discrepancy should be addressed in early discussions of data sharing between the food bank and healthcare system.

### Implementation facilitators

Across interviews with food banks and charitable food system experts, four facilitators needed for developing and sustaining food bank/healthcare partnerships were 1.) leadership support for addressing social determinants of health, 2.) mission compatibility/organizational readiness, 3.) food insecurity training, and 4.) identification of program/partnership champions.

All participants emphasized that need for leadership and staff at both the food bank and healthcare partner to have a strong desire to support all social determinants of health. Specifically, food bank leadership should have an interest in addressing other social determinants of health in addition to food access, and clinical partners should understand the interconnectedness of food insecurity with other social determinants of health. To address a variety of social determinants of health among food bank clients, participants suggested that food banks should participate in local health coalitions to collaborate with partner agencies and facilitate development of referral strategies.

The importance of mission compatibility and organizational readiness across both food banks and healthcare partners was also highlighted across all participants. Food banks must ensure that a food bank/healthcare partnership would further the food bank’s mission priorities, as it is possible that this kind of partnership may not be of interest or within scope for all food banks. Participants also reiterated that healthcare partners must have the willingness to treat food insecurity as an essential part of health and be willing to devote time and resources toward the partnership.

Food insecurity training was also discussed as a critical facilitators of food bank/healthcare partnerships. Participants felt it was essential for healthcare partners to receive training on the significance of food insecurity and how to successfully identify food insecure patients using the Hunger Vital Signs, a validated two-item food insecurity screening questionnaire based on the U.S. Household Food Security Survey Module to identify households at risk of food insecurity [20]. To support the partnership, participants also suggested that healthcare partners should universally screen all patients to ensure equitable access to program resources.

Program champions on both sides of a partnership was also discussed as a program facilitator. Within the food bank, there should be a partnership/program leader, as this person will be the point person for healthcare partnerships, decision making, and partnership outreach. Similarly, there is a necessity for a healthcare champion with dedicated time for the partnership that also holds leadership responsibility within the clinic, as this person will help facilitate partnership programs and ensure that the clinic upholds its responsibility.

## Discussion

Partnerships between charitable food systems and healthcare systems have the potential to provide support for at-risk communities through strategic support for both healthcare and emergency food relief. Study results outline unique implementation challenges for food bank/healthcare partnerships from the perspective of the charitable food system that are often not included in the literature discussing these food bank/healthcare partnerships. Results also outline implementation facilitators among food banks with existing food bank/healthcare partnerships.

Implementation challenges identified in this study can be organized into categories including partnership, program, and structural-level challenges. Partnership challenges are those that need to be discussed during formation of the partnership, as they hinge on the ability for both partners to work together efficiently and equitably. The most mentioned partnership-level implementation challenge was partnership/program scale. Scale was discussed in a few capacities including the number of participants that could be served, as well as the industrial-level scale that food banks operate most efficiently. This finding mirrors a recent review that suggested successful implementation of programs and partnership by food banks hinges on the ability to keep costs low [21], such that the program must be scaled to make the cost-benefit sustainable to the food bank.

The importance of data collection and evaluation were highlighted among implementation challenges. Results suggest that food banks and healthcare partners should invest time and energy into reaching an agreement on measurable outcomes that show meaningful success for both partners. This means that both food banks and healthcare must rethink traditional measures of success such as pounds of food distributed or clinical biometric health markers such as body mass index or hemoglobin A1c, a measure of long-term blood sugar control, as there are many other aspects of health. For example, it is reasonable to believe that food banks and healthcare partners would both value an outcome of increased healthcare provider self-efficacy for screening and referring patients with food insecurity to the local food bank, as this would allow providers to better serve their patients and food banks to serve additional clients. Differing measured outcomes across food bank/healthcare partnerships models also makes it challenging for evaluations across partnership types. A recent review found 23 published studies addressing food insecurity within the healthcare setting, yet few studies used the same methods or outcomes [22], making across study comparison difficult. Therefore, careful attention should be given to selecting meaningful measures that are relevant to both partners.

Another challenge in food bank/healthcare partnerships are expectations around food consistency and variety. This discrepancy centers on the inability of food banks to ensure consistent food products or produce at any given time of the year. While food banks have high purchasing power, their ability to purchase foods largely depends on funding and the ability to safely pack, transport, and store foods [23]. As noted by results, healthcare systems may have unrealistic expectations of what food banks are able to deliver. When expectations do not align with the availability of food products, it may be difficult for both charitable food systems and healthcare systems to continue to engage in a meaningful way.

In addition to partnership and program implementation obstacles, there will also likely be structural challenges such as food safety, federal regulations among food programs, and patient privacy. Food safety should be at the heart of any partnership that is planning for onsite food distribution, as there must be adequate dry and cold food storage in areas that meet food safety standards. The need for food safety and appropriate storage is echoed in a recent publication outlining the feasibility of healthy food pantries [24]. This structural challenge should not be discounted, as participants noted this can be a challenge for healthcare partners that want clinic-based food pantries. The complexities of federal regulations that specify how food bank purchased foods are used should also not be discounted, as this will often determine what food is available within a partnership. While challenging to navigate, this source of purchasing power should not be discounted, as it makes up a significant portion of a food banks funding. Lastly, results are consistent with previous work [25] suggesting that careful attention should be given to how to ensure patient privacy, while also allowing data sharing between partners.

Facilitators of food bank/healthcare partnerships documented in this study including leadership support, food insecurity training, and program champions are comparable to previous research findings among healthy food pantries [24]. With a focus on leadership support and program champions, results of this study also suggest that organizational readiness among food banks is important. Specific to these food bank/healthcare partnerships, attention should be given to the beliefs and attitudes of staff at food banks around their role to support health, not just hunger, as this may play a role in the relationship formation with healthcare. While food banks are moving to a nutrition focused mission, historically food banks were established to provide emergency food, with little attention to food quality. This transition from focusing to calories from food to focusing on nutrition from food is highlighted by the recent release of the first ever Nutrition Guidelines for the Charitable Food System [26].

Another study finding worth highlighting was focus on social determinants of health among food banks. Focusing on social determinants of health among food banks has increased in the past few decades given the vast research base linking food insecurity and other social determinants of health [27]. While the idea of charitable food systems functioning as a public health entity is a relatively novel idea, results of this study suggest it is top of mind for many food banks. This is evidenced by food banks emphasizing their role as partners in health promotion [25] and recent attention on incorporating social services teams into food banks [28]. Results also indicate that some food banks are actively participating in local health coalitions, suggesting they are interested in connecting resources and broadening their support of health within the community. Given that food banks are already creating spaces for social service discussions within their scope of work, it may be reasonable to build upon this foundation to further support public health priorities through charitable food systems, such as the vast network of food banks and pantries throughout the country.

While this study provides a unique perspective on partnerships between charitable food systems and healthcare, study limitations exist. This study was competed with a limited sample that largely represented a single state, yet many implementation barriers and facilitators should be similar regardless of where this partnership is taking place. This study was also completed as a pilot study with interview goals of exploring implementation barriers and facilitators, so structured questions were not asked of each participant nor were interviews recorded. Instead, interviews differed based on the participants experience and expertise and notes were taken. This method was chosen to provide the most comfort and privacy of participants while discussing barriers and allowing interviewer flexibility to explore new ideas, implementation barriers, and facilitators discussed by participants. To provide a more comprehensive understanding of food bank/healthcare partnerships, future research should consider a more systematic approach to understanding food bank/healthcare partnership models, logistics, and goals, as well as include perspectives from both food banks and healthcare partners.

## Conclusions

Ultimately, interviews with food bank experts suggest careful planning and implementation of food bank/healthcare partnerships can be formed to support the physical and social needs of individuals and families experiencing food insecurity. Attention should be given to implementation challenges both within the partnership and program, as well as structural challenges working across systems. To continue moving this work forward, a convening of leaders within charitable food systems and healthcare systems is needed to provide direction for partnerships, improve future collaborations, and create a more equitable implementation framework.

## Data Availability

The datasets used and/or analyzed during the current study are available from the corresponding author on reasonable request.

## Abbreviations

HHS: Health and Human Services
HIPAA: Health Insurance Portability and Accountability Act
